# Neuroprotection of exercise: P2X4R and P2X7R regulate BDNF actions

**DOI:** 10.1007/s11302-022-09879-x

**Published:** 2022-07-11

**Authors:** Bing-xin Sun, Ai-shi Peng, Pei-jie Liu, Min-jia Wang, Hai-li Ding, Yu-shi Hu, Liang Kang

**Affiliations:** 1 School of Sports Medicine and Health, Chengdu Sport University, Chengdu, 610041 China; 2 Institute of Sports Medicine and Health, Chengdu Sport University, Chengdu, 610041 China

**Keywords:** BDNF, Exercise, P2X4R, P2X7R

## Abstract

The neurotrophin brain-derived neurotrophic factor (BDNF), which acts as a transducer, is responsible for improving cerebral stroke, neuropathic pain, and depression. Exercise can alter extracellular nucleotide levels and purinergic receptors in central nervous system (CNS) structures. This inevitably activates or inhibits the expression of BDNF via purinergic receptors, particularly the P2X receptor (P2XR), to alleviate pathological progression. In addition, the significant involvement of sensitive P2X4R in mediating increased BDNF and p38-MAPK for intracerebral hemorrhage and pain hypersensitivity has been reported. Moreover, archetypal P2X7R blockade induces mouse antidepressant-like behavior and analgesia by BDNF release. This review summarizes BDNF-mediated neural effects via purinergic receptors, speculates that P2X4R and P2X7R could be priming molecules in exercise-mediated changes in BDNF, and provides strategies for the protective mechanism of exercise in neurogenic disease.

## Introduction

The neurotrophin brain-derived neurotrophic factor (BDNF) is a key mediator of neuroplastic changes induced by exercise. Physical activity has demonstrated positive effects, such as increased adult neurogenesis, in preventing and ameliorating a wide range of brain diseases. Disquietingly, it is possible to affirm that 46.6% of Parkinson’s disease (PD) patients in the Netherlands were less physically active since the COVID-19 lockdown, and reduced physical activity correlated with exacerbating PD symptoms (rigidity, fatigue, tremor, pain, and concentration) [1]. Encouraging or prescribing regular exercise can be worthwhile, due to major depressive disorder symptoms of low energy and motivation [2]. Meanwhile, adult-born neurons can migrate to regions of damage and facilitate repair with stroke, which can be further enhanced by exercise interventions [3]. In addition, repeated high-intensity swimming exercise reduces mechanical allodynia in complex regional pain syndrome type I mice [4]. Synthesis of BDNF is already the exact mechanism underlying exercise-induced neuroprotection in the hippocampus, hypothalamus, and cortex. This process plays crucial roles in gliogenesis, neurogenesis, synaptogenesis, and angiogenesis [5], leading to the enhancement of brain function [6]. The binding of BDNF to high-affinity tyrosine receptor kinase B (TrkB) can initiate at least 3 intracellular signaling pathways, including the mitogen-activated protein kinase/extracellular signal-regulated protein kinase (MAPK/ERK), phospholipase Cg (PLCγ), and phosphoinositide 3-kinase (PI3K) pathways [7, 8]. Regular exercise stimulated the expression of BDNF and TrkB in a stroke rat model, which can be negated by treatment with antisense BDNF oligonucleotide [9]. Treadmill exercise training increased BDNF and decreased Akt activity in the paraventricular nucleus post-myocardial infarction [10] and protected against IFN-α-induced decreases in the expression of BDNF in the hippocampus and prefrontal cortex [11]. In addition, treatment with a phosphoinositide-13 (PI3) kinase inhibitor reversed the beneficial effects of exercise-induced expression of BDNF in neurorepair [12].

**Fig. 1:**
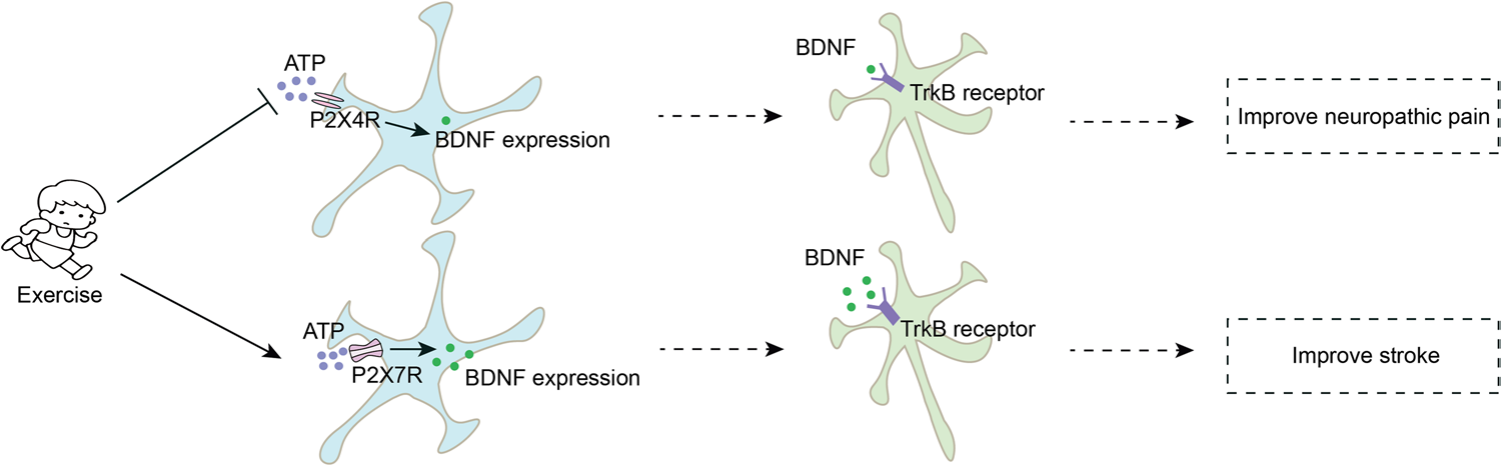
Hypothesis: Exercise could decrease the expression of BDNF by inhibiting P2X4R activation; exercise could increase the expression of BDNF by enhancing P2X7R activation. Exercise ameliorates neuropathic pain and stroke by regulating P2X4R and P2X7R activation. Eventually, P2X4R and P2X7R may complement neural effects due to the release of BDNF exercise

More evidence is still needed to clearly define the exercise-related BDNF mechanisms. On the other hand, the evidence supporting a role for exercise in purines has remained ambiguous. ATP was not only thought to be solely the universal energy currency but also in the nervous system is released into the extracellular space by neurons, astrocytes, and microglia through Panx1 channels to mediate inflammation and glial cell proliferation, which is engaged in the development of diseases such as stroke, epilepsy, and chronic pain [13]. Purinergic receptors (A1, A2A, A2B, P2X4, P2X7, P2Y11Rs) have been recognized as important mediators of BDNF activation and participate in multiple pathologies, including stroke, neuropathic pain, and depression (Table 1). Moreover, preconditioning exercise decreased P2X4 receptor (P2X4R) and BDNF, decreased inflammatory cytokines, and ceased prior to sciatic injury [14]. It has been reported that P2X7 receptor (P2X7R) and BDNF are probably involved in neuron structural and functional modifications that culminate in the enhancement of sensorimotor function after exercise [15]. Here, we provide support for the hypothesis that P2X4R and P2X7R activate the BDNF signaling pathway in the neuroprotection of exercise (Figure 1), which mainly contributes to anti-inflammatory and neuroplasticity.

**Table 1:** BDNF is regulated by P1/P2Rs

Purinergic receptors	Molecules	Functions	Effects	References
A2AR	BDNF↑	Inhibit GABA release, promote Glu release	\	[16]
		Enhance axonal elongation/dendritic branching		[17]
		Facilitate LTP on hippocampal CA1		[18]
	BDNF↑, pERK1/2↑	–	Improve global cerebral ischemia reperfusion injury	[19]
A1R	BDNF↑, pERK1/2↑	Decrease microglial reactivity	Improve global cerebral ischemia reperfusion injury	[19]
	BDNF↓	Decrease LTP on hippocampal CA1	–	[20]
P2X4R	BDNF↑, Iba1↑	Activate microglia	Exacerbate neuropathic pain	[21]
	BDNF↑, EAAT3↑	–	Exacerbate trigeminal allodynia	[22]
	BDNF↑, p38-MAPK		Improve cerebral hemorrhage-induced injury	[23]
P2X7R	BDNF↓	–	Exacerbate depression	[4, 24]
	BDNF↑		Exacerbate stroke pain	[25]
P2Y11R and A2BR	BDNF↑	–	Improve depression	[26]

↑ enhancement, ↓ decreased, – not determined

## Exercise improves neurological disorders by regulating BDNF and purinergic signaling

Physical exercise is a well-known and established method for the prevention and treatment of many diseases (metabolic syndrome, hypertension, neuronal and psychiatric disorders, among others) recognized by the American College of Sports Medicine (ACSM) [27–29]. Importantly, this includes powerful effects of endurance exercise on the brain and nervous system. As a striking example, a trial of endurance exercise training (moderate-intensity treadmill walking 3 days/week) was conducted in adults 55–80 years of age [30]. Participants in the exercise group exhibited a 2% increase in hippocampal volume that positively correlated with improvements in spatial memory. Moreover, running has been shown to fortify the organization of and communication within neuronal networks, which could have beneficial impacts on memory and spatial-temporal information processing [31]. Importantly, exercise-induced neuronal adaptations are accompanied by the proliferation of endothelial cells and angiogenesis in the cerebral cortex and hippocampus [32]. One mechanism through which acute/chronic physical exercise triggers responses/adaptations locally and globally is through the production and secretion of BDNF or other circulating factors, which beneficially upregulates stress-response pathways, induces vascularization, and promotes synaptic plasticity and neurogenesis [22, 33].

A group of researchers investigated the role of physical exercise in preventing and treating diseases and its relationship with purinergic signaling. Studies were carried out to investigate the modulation of the purinergic system (receptors, enzymes, and nucleotides) by physical exercise. Submitting rats to 20 min of a daily moderate treadmill for 2 weeks can significantly reduce adenosine triphosphate (ATP) and adenosine diphosphate (ADP) hydrolysis in the hippocampal synaptosomes and blood serum [23], suggesting lower activity of CD39. Possible modulation of A1R [4], A2AR, P2X2R, P2X6R [16], P2X4R [14], and P2X7R [15] by physical exercise was explored in complex regional pain syndrome type I (CRPS-I) mice, chronic constriction injury rats, stroke rats, and hypertensive rats. However, there is still a lack of information about the possible effects of physical training on some P2YRs.

## Purinergic receptors regulate BDNF

BDNF has been described to increase neuronal excitability and synaptic plasticity [17, 18], whereas the precursor of BDNF, proBDNF, preferentially binds to p75NTR and causes diametrically opposing effects, triggering apoptosis and synaptic depression [19, 20]. BDNF could promote the survival of serotonergic axons, enhancing 5‐HT uptake and its activity-dependent release. In mouse and postmortem sample patients, downregulation of BDNF was related to depression in various brain regions, including the anterior cingulate cortex, caudal brainstem, ventral prefrontal cortex, and hippocampus [21]. Intravenous BDNF injection after stroke could enhance neuronal remodeling, leading to improved functional motor recovery (rotarod, beam balance, neuroscore) [24]. The processes of sustaining chronic pain are also correlated with the sustained release of BDNF, while the maintenance of hyperalgesia has not been completely elucidated [26]. Emerging evidence indicates that A1, A2, and P2Rs are crucial regulatory factors in BDNF expression, which is essential for the release of glutamate vesicles and even microglial reactivity (Table 1). P2X4R and P2X7R are involved in neuropathic pain [34], intracerebral hemorrhage [35], and depression [36] by regulating BDNF actions.

It is accepted that P1 and P2Rs induce changes in BDNF expression in relevant CNS structures. A2AR activation is a determinant of the inhibitory actions of BDNF on GABA release and Glu release [37], the expansionary effects of BDNF on axonal elongation/dendritic branching [38] and the facilitation of LTP on hippocampal CA1 [39]. Hippocampal A1R or A2AR activation could elicit similar elevations in pERK1/2 in a model of global cerebral ischemia reperfusion injury, along with decreased microglial reactivity and increased BDNF expression via A1R [40]. However, a significant increase in BDNF levels was detected after caffeine application (a selective A1R antagonist) in hippocampal slices [41]. In vitro studies have also confirmed that BDNF production is dependent on P2X4R using an ATP-activated spontaneously immortalized microglial cell line [42]. Genetic deletion of P2X7R appears to be the cause of the increase in BDNF [43]. ATP exocytosis induced by the antidepressant fluoxetine increased the astrocytic expression of BDNF mRNA in primary cultures of hippocampal astrocytes, which was mediated by activation of P2Y11R and A2BR. Moreover, intracellular signaling cascades of BDNF release are associated with the accumulation of cAMP and the activation of protein kinase A (PKA), not Ca^2+^/calmodulin-dependent kinase (CaM kinase) [44]. However, some evidence indicated that ATP-triggered vesicle fusion and BDNF secretion were probably related to astrocytic Ca^2+^ excitability [45].

## Exercise alleviates neuropathic pain by inhibiting the P2X4R-BDNF pathway

Typically, neuropathic pain is characterized by hyperexcitability of the primary afferent sensory neurons accompanied by the release of transmitters or mediators such as BDNF [46] and glutamate [47]. The increased mBDNF/proBDNF ratio mediated by ATP administration was dependent on P2X4R, which was confirmed by P2X4R-shRNA treatment [35]. The communication of microglial P2X4R and neurons is an essential link in pain transmission, and BDNF-TrkB signaling also plays a crucial role in analgesia. Notably, the upregulation of excitatory amino acid transporter 3 (EAAT3), a subtype of sodium-dependent EAATs, was accompanied by increased P2X4R and BDNF in the microglia of rats following trigeminal allodynia induced by repeated dural IS infusions [34]. Moreover, the activated P2X4R in microglia causes the phosphorylation of p38-MAPK, resulting in the release of BDNF, all of which are essential to the persistence of pain hypersensitivity after nerve injury. Therefore, it seems plausible that blockade of the P2X4R-p-p38-MAPK-BDNF pathway in the spinal cord may provide a novel therapeutic strategy for neuropathic pain [25].

Growing evidence suggests that exercise alleviates neuropathic pain in rats. Six weeks of voluntary rotation before the onset of chronic compression injury prevented the full development of ectopic pain, normalized the expression of excitatory interleukin (IL)-1β, and decreased the expression of the P2X4R-BDNF axis in the dorsal spinal cord of the ipsilateral spinal cord [14]. High-intensity exercise caused a significant adenosine concentration in the entire rat brain. In the subsequent sleep period, adenosine levels decrease, and the concentrations of ADP and ATP increase [48]. Low and high exercise frequencies both significantly prevented A2AR overactivation [49]. The changes in purines after exercise observed in experiments support that ATP and nucleotides are able to interact with BDNF to amplify or modulate their signaling, resulting in amelioration of neuropathology.

P2X4R expression in microglia and the subsequent release of BDNF are both required for hyperalgesia. Specifically, P2X4R activation by ATP at the cell surface leads to Ca^2+^ influx, phosphorylation of p38, and synthesis and release of BDNF, causing disinhibition of nociceptive projection neurons [50]. When P2X4R/Trk-Fc was blocked, inhibition of HSV-1-induced allodynia was triggered [51]. Basically, BDNF can protect neurons against various insults [9, 10] and the neuroprotection of P2X4R after stroke by promoting the synthesis of mBDNF [35]. Moreover, P2X4R upregulation does not occur based on any exercise modalities. Interestingly, preconditioning exercise decreased P2X4R and BDNF, ceased prior to sciatic injury, and decreased neuroimmune signaling in the spinal dorsal horn [14]. Furthermore, in relation to other P2XRs, a high ATP concentration does not activate P2X4R until the pH increases to 7.4 [51]. Therefore, it is highly likely that P2X4R will not be activated to mediate BDNF synthesis during regular or vigorous exercise because H^+^ production has traditionally been explained by the exercise-induced increased production of lactic acid [52].

## Exercise improves stroke probably by activating P2X7R and BDNF

Brain expression of P2X7R has been described in all intrinsic cells of the CNS, and functional P2X7R and downstream signaling pathways are extremely debated. Previous observations have described that longer C-terminal domains of P2X7R are responsible for the activation of downstream signaling pathways, including the ERK pathway and activation of caspase-3, resulting in the initiation of apoptosis [53, 54]. Generally, P2X7R has detrimental and beneficial roles in the nervous system. On the one hand, evidence strongly indicates that excessive P2X7R activation is involved in the caspase-independent death of neural progenitor cells [55]. In particular, prolonged activation of P2X7R is thought to be largely associated with pathological conditions where the extracellular ATP concentration rises dramatically due to inflammation or leakage from cell damage [56]. Accordingly, the involvement of P2X7R during the pathogenesis of multiple chronic disorders of the CNS has been demonstrated [57, 58]. On the other hand, P2X7R stimulation with low amounts of Bz-ATP in rat embryonic NPCs induced neuronal differentiation rather than cell death [59]. Meanwhile, BDNF plays a crucial role in the development, differentiation, and survival of neuronal populations within the central and peripheral nervous systems. In relation to inflammation, there is no definite mechanism for the effects of proinflammatory cytokines on the expression of BDNF, whereas proinflammatory cytokines result in a significant reduction in BDNF [60].

Traditionally, the expression of P2X7 is negatively correlated with BDNF. The P2X7R antagonist A-804598 (3, 10, or 30 mg/kg/day) was used to investigate BDNF signaling in the rat frontal cortex and ventral and dorsal hippocampus. The results showed that antagonizing P2X7R may activate TrkB and mediate an increase in BDNF-AKT-p70 S6 kinase levels in the ventral hippocampus, which produces a related antidepressant effect [36]. Elevated basal BDNF levels and neurogenesis in the hippocampus of P2X7R^−/−^ mice compared with P2X7R^+/+^ controls indicate tonic inhibitory regulation of BDNF production through P2X7R [43]. In line with this argument, experimental models have demonstrated that inhibition of P2X7R alleviates depression via BDNF activation [36]. Unfortunately, pharmacological antagonists of P2X7R have been produced and tested in various diseases with mostly disappointing results [61, 62]. Another study, however, suggests otherwise. Central poststroke pain (CPSP) was used to examine continuous sensitization behavior in rats, which was suppressed by a P2X7R antagonist and BDNF receptor blocker. The results demonstrated that treatment of stroke pain with a P2X7R antagonist can prevent microglial P2X7R activation in the surrounding areas of CPSP lesions and reduce the release of regional inflammatory cytokines [63]. More importantly, Glu release is an important function of P2X7R activation related to BDNF expression; in turn, subsequent overactivation of extrasynaptic NR2B receptors (NMDA receptor subunits) downregulates BDNF expression [43]. It has been reported that P2X7R controls BDNF release from Schwann cells, which exert neuroprotective effects on neighboring vestibular neurons [64]. Therefore, negative feedback loops of molecular interaction (P2X7R and BDNF) in cells may be the main mechanism of disease and therapy. In addition, considering that P2X7R cooperates with NMDA and BDNF receptors to promote neuronal survival through both the PKC and PI3K/Akt pathways, a precise interpretation of the results is that P2X7R and BDNF play neuroprotective roles.

Interestingly, preconditioning exercise significantly reduced infarct volume and apoptosis, in which P2X7R and BDNF in the ischemic brain were significantly upregulated. Following intracerebral hemorrhage, ATP administration had the ability to relieve cerebral hemorrhage–induced injury and improve cerebral neurological function by upregulating the mBDNF/proBDNF ratio and p38-MAPK. However, whether P2X7R has a clear regulatory effect on BDNF has not been clearly demonstrated in the area of stroke improvement [15].

## Conclusion

Owing to the relatively few discussions of the purinergic constituents in neuroprotection during or after exercise, it is pressing to explore their physiology. As discussed in this review, some P1/P2Rs have been recognized as significant mediators of BDNF expression, which participate in multiple pathologies, including stroke, neuropathic pain, and even depression. Both P2X4R and P2X7R influence BDNF activation, leading to accumulation of p38-MAPK and upregulation of Iba1 and pERK1/2. A1, A2A, A2B, P2X4, P2X7, and P2Y11Rs have been shown to participate in regulating BDNF expression. However, interactions between the downstream signaling mechanisms of P2X7R may also occur. Eventually, P2X4R and P2X7R may complement neural effects due to the release of BDNF by exercise.

## Data Availability

Not applicable.

## Abbreviations

ATP: adenosine triphosphate
ADP: adenosine diphosphate
CNS: central nervous system
TrkB: tyrosine receptor kinase B
MAPK: mitogen-activated protein kinase
ERK: extracellular signal-regulated protein kinase
PI3K: phosphoinositide 3-kinase
PLCγ: phospholipase Cg
EAAT3: excitatory amino acid transporter 3
CPSP: central post stroke pain
PD: Parkinson’s disease
Glu: glutamic acid
GABA: gamma-aminobutyric acid
—R: receptor
